# Management of Severe Burn Microstomia

**Published:** 2016-11-22

**Authors:** Megan Zak, Olivia Means, Benton Cason, Ron Brooks

**Affiliations:** ^a^University of South Alabama College of Medicine, Mobile; ^b^Department of Surgery, Plastic and Reconstructive Surgery, Universtiy of South Alabama Medical Center, Mobile

**Keywords:** microstomia, burn, commissuroplasty, contracture, reconstruction

## DESCRIPTION

**Figure 1:**
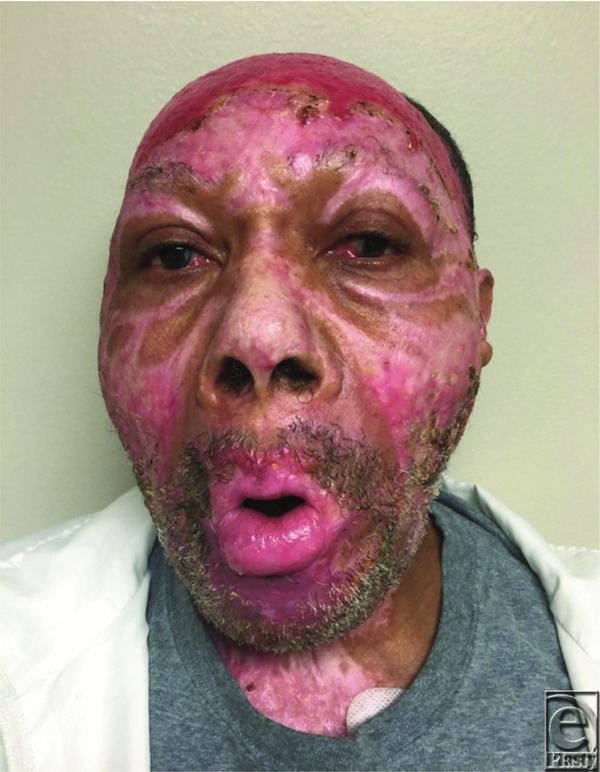
Bilateral oral commissure contractures and resulting microstomia 7 months postburn. Interlabial distance 5 mm.

A 67-year-old man developed severe microstomia caused by bilateral scar contractures as a result of flame burn injuries sustained to the head and neck regions. Preoperative interlabial gap measured 5 mm (Fig 1) and interfered with enteral intake, communication, and blunted his affect. He underwent bilateral commissuroplasty after nonoperative splinting therapy failed.

## QUESTIONS

1. **What are the etiology and sequelae of microstomia?**
2. **Describe nonoperative management of microstomia.**
3. **Describe operative management of microstomia.**
4. **What is the prognosis following surgical management of microstomia?**

## DISCUSSION

Microstomia describes congenital or acquired reduction of the oral aperture due to contracture of the lateral commissures. The most significant congenital syndrome associated with microstomia is Freeman-Sheldon syndrome,^1^ but most cases are acquired. Acquired etiologies include ingestion of caustic substances,^2^ trauma, oral-facial burns, previous lip reconstruction, and connective tissue disorders, such as scleroderma.^3^ Sequelae of microstomia include, but are not limited to, malnutrition, respiratory and speech difficulties, risk of aspiration, poor oral hygiene, difficulty with future endotracheal intubation, and cosmetic concerns.^2^ As a result of his severe microstomia, our patient was able to eat only pureed foods, avoided conversation due to speech difficulty, and had a blunted affect from his limited ability to communicate and from appearance of his face, especially his mouth. Despite his pureed diet limitations, preoperative testing did not reveal any nutritional deficiencies.

Perioral facial burns have a high propensity for scar contractures due to the circumferential nature of the orbicularis oris muscle. Nonoperative management is the preferred initial therapy for perioral and facial burns, given the face's ability to heal secondary to its robust blood supply. Splinting is the most common therapy and is usually supplemented with physical therapy, oral exercises, and massage. There are a variety of extra- and intraoral splinting devices that are chosen on the basis of the patient and nature of the injury. While there continues to be debate over the optimal management and timeline for facial burns, there is consensus that use of splinting decreases the likelihood for later surgical reconstruction. Noncompliance is the most common cause for failure of nonoperative management and need for subsequent surgery,^4^ as was the case in our patient.

**Figure 2:**
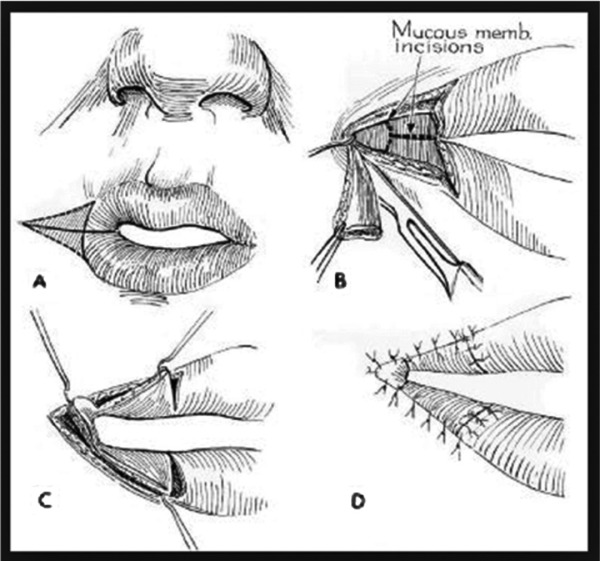
Y-V mucosal advancement flaps originally described by Dieffenbach, with later modifications by Converse.

**Figure 3:**
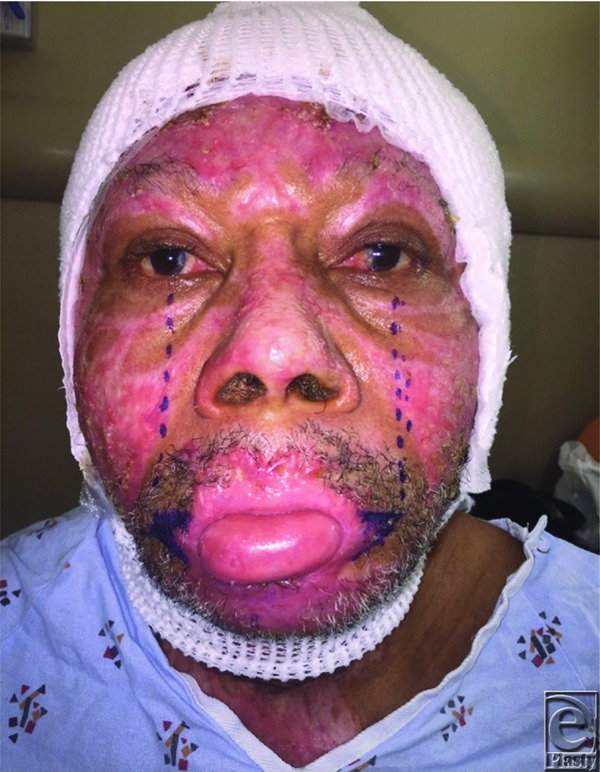
Preoperative markings. Mid-pupillary axis used to determine new lateral oral commissure location.

**Figure 4:**
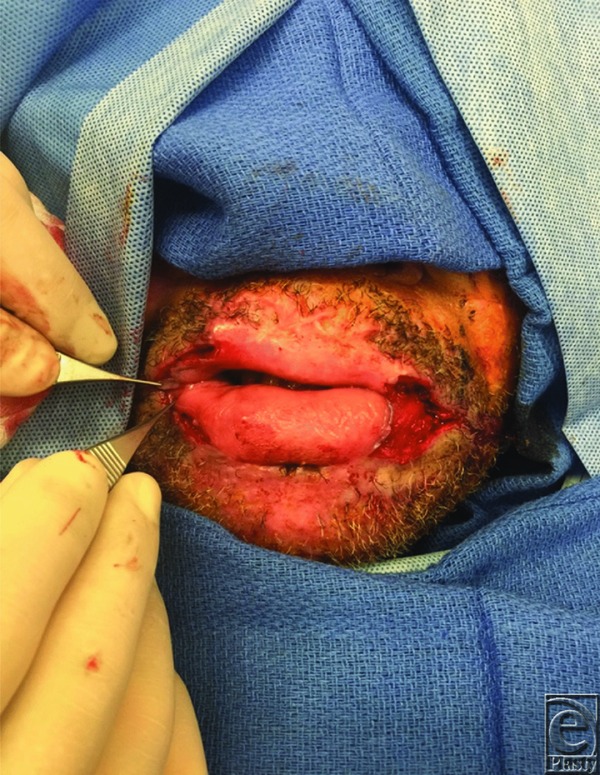
Intraoperative view of buccal mucosa flaps. Bilateral Y-V advancement flaps following burn scar excision.

Dieffenbach first established a technique in 1831 to treat microstomia, which involved a Y-V advancement of superior, inferior, and lateral mucosal flaps after a wedge-shaped excision of the scar.^5^ Modifications have since been introduced by Converse,^6^ Kazanjian and Roopenian,^7^ and others, who describe a combination of a vermillion advancement or buccal mucosal transposition (Fig 2).^2^ Alternative surgical options include z-plasties, rhomboid flaps, and scar excision with full-thickness or split-thickness skin grafts.^8^ We used the technique described by Dieffenbach to reconstruct the commissures bilaterally. Using the mid-pupillary line as a landmark for the lateral commissure (Fig 3), we performed a triangular wedge skin excision and burn scar release, preserving the orbicularis oris muscle. We then developed a plane between the posterior orbicularis muscle and the buccal mucosa to allow creation and advancement of the mucosal flap. A Y-shaped incision was created to form 3 separate mucosal flaps. The central flap was closed to the acute angle of the oral commissure, followed by advancement of the remaining 2 flaps to reconstruct the lateral upper and lower lips (Fig 4).

Following commissuroplasty, complications include oral incompetence secondary to orbicularis muscle injury or dysfunction, tongue or lip adhesions, recurrent contracture, and even carcinoma from chronic burn scar.^2^ Optimal prognosis hinges on preserving the reconstructed oral aperture through splinting and mouth therapy, which will decrease risk of recurrence and other complications. Oral commissure reconstruction improves oral intake and nutrition, as well as other associated difficulties with speech, respiration, and cosmesis.^5^

**Figure 5:**
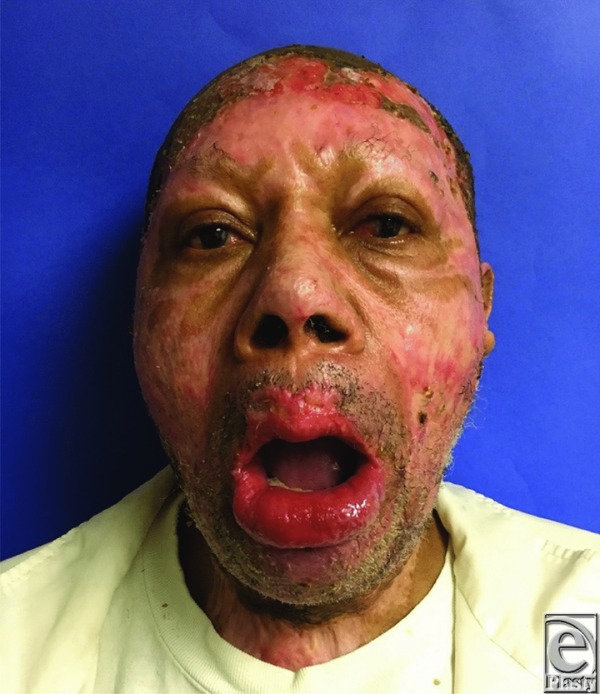
Postoperative view at 5 weeks, with interlabial distance 2.7 cm.

Postoperatively, our patient has been much more compliant with his speech therapy and splinting as opposed to after his initial burn injury. He is undergoing balloon catheter buccal dilation for a total of 1 hour per day, as well as multidirectional splinting to maintain and improve his oral aperture. At 5 weeks, his interlabial gap measures 2.7 cm (Fig 5), and he reports enjoying eating again with increased enteral intake from a variety of solid foods. In addition, his affect has improved considerably and his family reports he is much more verbal than prior to his commissuroplasty.
